# Inter occasion variability in individual optimal design

**DOI:** 10.1007/s10928-015-9449-6

**Published:** 2015-10-01

**Authors:** Anders N. Kristoffersson, Lena E. Friberg, Joakim Nyberg

**Affiliations:** Department of Pharmaceutical Biosciences, Uppsala University, Box 591, 75124 Uppsala, Sweden

**Keywords:** Inter occasion variability (IOV), Optimal design (OD), Maximum a posteriori (MAP), Fisher information, Bayesian, Pharmacometrics, Shrinkage

## Abstract

Inter occasion variability (IOV) is of importance to consider in the development of a design where individual pharmacokinetic or pharmacodynamic parameters are of interest. IOV may adversely affect the precision of maximum a posteriori (MAP) estimated individual parameters, yet the influence of inclusion of IOV in optimal design for estimation of individual parameters has not been investigated. In this work two methods of including IOV in the maximum a posteriori Fisher information matrix (FIM_MAP_) are evaluated: (i) MAP_occ_—the IOV is included as a fixed effect deviation per occasion and individual, and (ii) POP_occ_—the IOV is included as an occasion random effect. Sparse sampling schedules were designed for two test models and compared to a scenario where IOV is ignored, either by omitting known IOV (Omit) or by mimicking a situation where unknown IOV has inflated the IIV (Inflate). Accounting for IOV in the FIM_MAP_ markedly affected the designs compared to ignoring IOV and, as evaluated by stochastic simulation and estimation, resulted in superior precision in the individual parameters. In addition MAP_occ_ and POP_occ_ accurately predicted precision and shrinkage. For the investigated designs, the MAP_occ_ method was on average slightly superior to POP_occ_ and was less computationally intensive.

## Introduction

Inter occasion variability (IOV) is increasingly quantified in nonlinear mixed effect (NLME) models, but the impact of this type of variability on the optimal experimental design (OD) for the estimation of individual parameters is not clear. The NLME approach splits the model in fixed effects describing the typical population value parameters and different levels of random effects. Typically in pharmacokinetic (PK) and pharmacodynamic (PD) analyses inter individual variability (IIV) and residual error (RE) are estimated, but if variability between occasions (e.g. between dosing occasions or observation periods) is apparent IOV could be introduced as a third level of random effects [1].

With a Bayesian approach individual and occasion deviations from the typical population parameters can be estimated given a population model, its population parameter estimates, and individual observations. Individual parameter estimates, referred to as *Empirical Bayes Estimates* (EBEs), can be derived by *Maximum a Posteriori* (MAP) estimation and are of interest in e.g. model diagnostics [2], covariate analysis [3, 4] and feedback dose individualization [5]. Good precision of the EBEs are therefore of importance for effective model evaluation and for understanding and determination of individual differences in PK and PD. Characterization of individual parameters can also be of importance for establishing concentration-effect relationships [6]. If little information is provided about the individual parameters the patient will be regarded as a typical representative of the population and the predicted EBEs will be close to the typical population predictions, an effect known as η-shrinkage [2]. Conversely, if the individual information is rich the prior population information will have smaller influence and the predicted EBEs will be closer to the “true” individual values. The information richness of an individual may be improved by increasing the quantity of samples or by increasing the quality per sample, e.g. by optimal design (OD) methodology [7].

In OD a design criterion is used to link the experimental design to the measure of interest, commonly the joint precision of the parameter estimates. The determinant of the maximum a posteriori Fisher Information Matrix (FIM_MAP_), first suggested for NLMEs by Merlé and Mentré in 1995 under the name *Bayesian Information Matrix* [8], may be used as optimization criterion for individual deviation EBEs (henceforth called η_EBE_) [9, 10]. The FIM_MAP_ is the expectation of the individual FIM over the IIV distribution with the population distribution as prior information. The FIM_MAP_ follows the Cramér-Rao inequality so that its inverse is the lower bound for the expected posterior covariance matrix of an unbiased estimator of the individual parameters [8]. Hence the expected posterior covariance matrix of the η_EBE_s may be minimized by maximizing the inverse FIM_MAP_. Two additional metrics has been proposed to more closely follow the true posterior covariance matrix than the FIM_MAP_: the expected information provided by the experiment, and the pre-posterior covariance matrix (familiarly obtained through e.g. stochastic simulation and estimation) [8]. While the expected information provided by the experiment has been employed for design optimization [11], these methods are considerably more computationally demanding compared to the FIM_MAP_ [8, 12] and will not be further considered in this work.

Even though IOV has long been recognized to be of importance in NLME and neglecting IOV may negatively affect the precision of MAP estimated η_EBE_ [1], the inclusion of IOV has not been previously investigated for individual OD in a NLME framework. This work aims to evaluate possible design criteria permitting OD for individual parameter estimates in the presence of IOV. As a driving example the design of a study (AIDA) aimed at correlating individual PK of the antibiotic colistin with patient covariates and treatment outcome is used (www.aida-project.org). The trial will include over 300 patients and a sparse sampling design was to be suggested. Colistin was first used in the fifties but was later abandoned due to toxicity concerns and hence sufficient exposure–response information is missing. During recent years colistin has seen resurgent use in treatment of multi drug resistant gram negative infections [13]. A recent PK model for colistin and its prodrug colistimethate sodium (CMS) by Mohamed et al. [14] has quantified pronounced IOV in the PK parameters and this model was considered for MAP estimation of individual parameters from the AIDA study. The OD of a sampling schedule was however hampered by the current lack of methods to handle the IOV contributions in the model. The model has a complex random effects structure and a combined residual error model and the colistin PK model will thus serve as a complex example of MAP optimization in the presence of IOV. In addition to the colistin PK model a simple constructed 1-compartment IV-bolus population PK model with an additive or combined (additive plus proportional) residual error model (1-COMP) will be employed as a simpler test case.

Two possible methods to include IOV in the FIM_MAP_ were explored: (i) MAP_occ_ where the IOV is included as an individual deviation per occasion and individual, and (ii) POP_occ_ where the IOV is included as an occasion random effect. These methods were compared against two cases ignoring IOV, (i) Omit where the known IOV was omitted from the FIM_MAP_, and (ii) Inflate mimicking a situation where the study design neglected the possibility to quantify IOV, e.g. by placing all samples in one occasion within an individual. The methods were evaluated in terms of η_EBE_ precision (measured by simulation and MAP re-estimation) and estimation run-times for discrete designs with fixed sampling times (i.e. the same design for all individuals). We also considered the correspondence between predicted and evaluated precisions in η_EBE_ as well as the ease of use of the evaluated methods. Recently a method to predict shrinkage in the distribution of η_EBE_ from the FIM_MAP_ was presented by Combes et al. [15]. The ability of this method to predict shrinkage from the FIM_MAP_ with the proposed additions was here evaluated as a secondary objective. To accurately predict the precision and shrinkage of the η_EBE_ is of value as it would allow design appraisal without secondary simulation based methods.

## Methods

### Model structure

The test models used in this work are NLME models where the ith vector of individual responses **y**_i_ is defined as:

1 $$\varvec{y}_{\text{i}} = {\text{f}}\left( {{\varvec{\upchi}}_{\text{i}} , {\text{g}}\left( {{\varvec{\uptheta}}, {\varvec{\upeta}}_{\text{i}} , {\varvec{\upkappa}}_{{1,{\text{i}}}} , {\varvec{\upkappa}}_{{2,{\text{i}}}} \ldots ,{\varvec{\upkappa}}_{{{\text{m}},{\text{i }}}} } \right)} \right) + {\text{h}}\left( {{\varvec{\upchi}}_{\text{i}} , {\text{g}}\left( {{\varvec{\uptheta}}, {\varvec{\upeta}}_{\text{i}} , {\varvec{\upkappa}}_{{1,{\text{i}}}} , {\varvec{\upkappa}}_{{2,{\text{i}}}} \ldots , {\varvec{\upkappa}}_{{{\text{m}},{\text{i }}}} } \right),\varvec{ } \epsilon_{i} } \right)$$

where g() is the vector function describing the parameters for the ith individual defined by the typical population parameter vector ${\varvec{\uptheta}} = \left\{ {\theta_{1} , \theta_{2} , \ldots \theta_{d} } \right\}$, the individual deviations vector ${\varvec{\upeta}}_{i} = \left\{ {\eta_{1} , \eta_{2} , \ldots \eta_{u} } \right\} \sim {\text{N}}\left( {0, {\varvec{\Omega}}} \right),$ and the *m* occasion deviation vectors ${\varvec{\upkappa}}_{x,i} = \left\{ {\kappa_{1} , \kappa_{2} , \ldots \kappa_{v} } \right\} \sim {\text{N}}\left( {0, {\varvec{\Pi}}} \right)$. f() describe the structural model dependent on the individual design given by ${\varvec{\upchi}}_{\text{i}}$ and h() is the error model dependent on the residual error deviation vector $\epsilon_{i} \sim {\text{N}}\left( {0,\varvec{ }{\varvec{\Sigma}}} \right)$. The Matrices **Ω**, **Π**, and **Σ** describe the covariances of the individual, occasion and residual error deviations respectively. In this work all individuals were set to have the same elementary design, i.e. ${\varvec{\upchi}}_{\text{i}} = {\varvec{\upchi}}$, although this is not necessary within this framework.

### Colistin PK

The PK model applied for colistin and its prodrug CMS consists of one compartment for the formed colistin (Col) and two compartments for CMS, i.e. the central (CMS1) and peripheral (CMS2) compartments [14]. Parameter values are presented in Table 1. The structural model is described by the differential equation system:

2 $$\frac{{dA_{CMS1} }}{dt} = A_{CMS2 } (t)\frac{{\theta_{Q} e^{{\eta_{Q} }} }}{{\theta_{{V_{CMS2} }} e^{{\kappa_{{V_{CMS2} }} }} }} - A_{CMS1 } (t)\frac{{\theta_{Q} e^{{\eta_{Q} }} }}{{\theta_{{V_{CMS1} }} }} - A_{CMS1 } (t)\frac{{\theta_{{CL_{CMS} }} e^{{\eta_{CL} + \kappa_{{CL_{CMS} }} }} }}{{\theta_{{V_{CMS1} }} }}$$

3 $$\frac{{dA_{CMS2} }}{dt} = A_{CMS1 } (t)\frac{{\theta_{Q} e^{{\eta_{Q} }} }}{{\theta_{{V_{CMS1} }} }} - A_{CMS2 } (t)\frac{{\theta_{Q} e^{{\eta_{Q} }} }}{{\theta_{{V_{CMS2} }} e^{{\kappa_{{V_{CMS2} }} }} }}$$

4 $$\frac{{dA_{Col} }}{dt} = A_{CMS1 } (t)\frac{{\theta_{{CL_{CMS} }} e^{{\eta_{CL} + \kappa_{{CL_{CMS} }} }} }}{{\theta_{{V_{CMS1} }} }} - A_{Col } (t)\frac{{\theta_{{CL_{Col} }} e^{{\eta_{CL} \times \theta_{sc} + \kappa_{FM} }} }}{{\theta_{{V_{Col} }} e^{{\kappa_{FM} }} }}$$

where *A*_x_ is the drug amount, *CL*_x_ the clearance (CL/fm for colistin) and *V*_x_ the volume of compartment *x* (V/fm for colistin). The CL of colistin and CMS1 are 100 % correlated and share the common IIV random effect *η*_*CL*_, with *θ*_*sc*_ scaling the difference in the magnitude of IIV. *Q* is the intercompartmental clearance between compartments CMS1 and CMS2, and *fm* the fraction of CMS metabolized to colistin. The initial condition for all compartments is zero and the dosing compartment is *A*_*CMS1*_.

**Table 1:** Parameter values for the population PK model of colistin and CMS by Mohamed et al. [14]

Parameter (units)	Explanation	CMS					Colistin				
		Typical value	IIV		IOV		Typical value	IIV		IOV	
			CV %	Ω	CV %	Π		CV %	Ω	CV %	Π
CL_CMS_ or CL_col_(L/h)	Clearance of CMS or formed colistin	13.1	42 (45)	0.17 (0.20)	30 (0)	0.09 (0)	8.2	76 (81)^a^	0.61 (0.70)^a^	48 (0)	0.09 (0)
V_CMS1_ or V_col_(L)	Volume of distribution of central compartment for CMS or formed colistin	11.8					218			48 (0)	0.35 (0)
Q (L/h)	Intercompartmental clearance for CMS	206	111 (181)	1.23 (3.26)							
V_CMS2_ (L)	Volume of distribution in peripheral compartment for CMS	28.4			59 (0)	0.35 (0)					
Additive residual error (μM)		0.071	52 (135)	0.27 (1.8)			0.044				
Proportional error (%)		23	52 (135)	0.27 (1.8)			8.2				

Values in parenthesis are the IIV random effects inflated through model reestimation to include IOV. The variance parameters are presented both as coefficient of variation (CV %—left) of the typical value and as variances (Ω or Π—right)

^a^The IIV of CL_col_ is scaled from the IIV of CL_CMS_ by the scaling factor θ_sc_ = 1.81 on standard deviation scale

The combined additive and proportional residual error model includes IIV on the residual error of CMS allowing the residual error variance to differ between individuals. The dependent variables are the log transformed central compartment concentration of CMS (DV_CMS1_) and colistin concentration (DV_Col_) given in Eqs. 5, 6:

5 $$DV_{CMS1} \left( t \right) = \ln \left( {C_{CMS1} \left( t \right)} \right) + \epsilon \times e^{{\eta_{ER} }} \times \sqrt {\theta_{{ER_{CMS,prop} }}^{2} + {\raise0.7ex\hbox{${\theta_{{ER_{CMS,add} }}^{2} }$} \!\mathord{\left/ {\vphantom {{\theta_{{ER_{CMS,add} }}^{2} } {\ln \left( {C_{CMS1} \left( t \right)} \right)^{2} }}}\right.\kern-0pt} \!\lower0.7ex\hbox{${\ln \left( {C_{CMS1} \left( t \right)} \right)^{2} }$}}}$$

6 $$DV_{Col} \left( t \right) = \ln \left( {C_{Col} \left( t \right)} \right) + \epsilon \times \sqrt {\theta_{{ER_{Col,prop} }}^{2} + {\raise0.7ex\hbox{${\theta_{{ER_{Col,add} }}^{2} }$} \!\mathord{\left/ {\vphantom {{\theta_{{ER_{Col,add} }}^{2} } {\ln \left( {C_{Col} \left( t \right)} \right)^{2} }}}\right.\kern-0pt} \!\lower0.7ex\hbox{${\ln \left( {C_{Col} \left( t \right)} \right)^{2} }$}}}$$

where $C_{CMS1} \left( t \right) = \frac{{A_{CMS} \left( t \right)}}{{\theta_{{V_{CMS1} }} }}$, and $C_{Col} \left( t \right) = \frac{{A_{Col} \left( t \right)}}{{\theta_{{V_{Col} }} e^{{\kappa_{fm} }} }}$. The residual error variance was fixed to one and scaled by a proportional part given by *θ*_*ERx,pro*p_, and an additive part given by *θ*_*ERx,add*_, with the inter-individual variability described by *η*_*ER*_ for the CMS1 residual error.

For the Inflate case the IIV was inflated to accommodate the IOV in order to mimic a scenario where insufficient information to separate IIV and IOV caused all IOV to end up in the IIV (e.g. all samples taken in only one occasion). For the Colistin PK model by Mohamed et al. [14] (implemented in NONMEM 7 [16] with the ADVAN5 solver and FOCEI method) this was accomplished by removing the IOV random effects and fixing the typical population parameters (including the residual error parameters). The model was then rerun on the original dataset and only the IIV variances were estimated forcing the IOV variance into the IIV. The new IIV matrix was taken as Ω*. The IIV parameters for the Inflate case are presented along with the original parameters in Table 1. For simplicity and comparability with the 1-COMP model the residual error was not allowed to inflate.

In the clinical study a dosing regimen of 9MU (413 µmol) CMS as load (30-min infusion), followed by a maintenance dose of 4.5MU (30-min infusion) every twelfth h (q12) is planned to be administered. One occasion was defined as one dose interval, similar as in the model development [14].

### 1-COMP

The 1-compartment IV-bolus structural model is defined by the differential equation:

7 $$\frac{dA}{dt} = - A \times \frac{{\theta_{CL} e^{{\eta_{CL} + \kappa_{CL} }} }}{{\theta_{V} e^{{\eta_{V} + \kappa_{V} }} }}$$

where *A* is the drug amount, *V* is the volume of distribution and *CL* is the drug clearance. An additive or additive and proportional (combined) residual error model was used. The parameter values are found in Table 2. A q6 dosing regimen given as 1 unit IV bolus was implemented with one occasion per dose interval (6 time units).

**Table 2:** Parameter values for the constructed 1-COMP model

Parameter	Typical value	IIV (%)		IOV (%)	
		CV %	Ω	CV %	Π
CL	9	25 (35)	0.0625 (0.125)	25 (0)	0.0625 (0)
V	40	25 (35)	0.0625 (0.125)	25 (0)	0.0625 (0)
Additive residual error variance	1				
Proportional residual error variance	0.04				

Values in parenthesis are the IIV random effects inflated to include IOV. The variance parameters are presented both as coefficient of variation (CV %—left) of the typical value and as variances (Ω or Π—right)

As the IIV and IOV are included on the same parameters the IOV inflated IIV, Ω*, for case Inflate was taken as the sum of the IIV and IOV variances so that *ω*_r_^2^* = *ω*_r_^2^ + *π*_r_^2^, where *ω*_r_^2^* is the rth diagonal element of Ω*. The original values and the values for scenario Inflate can be found in Table 2.

### FIM_MAP_

The approximation of the FIM_MAP_ and the notation used was based on the work by Hennig et al. [10]. Here we give a brief description of the procedure, for a detailed description please see Merle and Mentre [8].

In order to calculate the FIM_MAP_ the population model was transformed to an individual model transferring the population random effect parameters, **η**, to individual parameters, **θ**_*η*_, sampled from **Ω**. The process is described in Eq. 8:

8 $$\textbf{p}_{\text{i}} = g\left( {{\varvec{\uptheta}}, {\varvec{\upeta}}_{\text{i}} } \right) \to g\left( {{\varvec{\uptheta}}_{\theta } , {\varvec{\uptheta}}_{{\eta_{\text{i}} }} } \right), {\varvec{\uptheta}}_{{\eta_{\text{i}} }} \sim N\left( {0,{\varvec{\Omega}}} \right)$$

where **p**_i_ is the parameter vector for individual i dependent on the population parameters **θ**_*θ*_, and the individual parameters **θ**_*ηi*_.

The FIM_MAP_ is formed as the expectation of the individual FIM for the transformed model over its prior, ${\varvec{\Omega}}^{ - 1}$. The expectation was here approximated by Monte Carlo integration over all possible individual parameter values. The procedure is given by:

9 $$\begin{array}{*{20}l} {{\mathbf{FIM}}_{\text{MAP}} = {\text{E}}_{{{\mathbf{Prior}}}} \left[ {{\mathbf{FIM}}_{{\mathbf{i}}} } \right] + {\mathbf{Prior}} \approx \frac{1}{n} \times \mathop \sum \limits_{i = 1}^{n} {\mathbf{FIM}}_{{\mathbf{i}}} + {\mathbf{Prior}}} \\ {\text{where:}} \\ { {\mathbf{FIM}}_{{\mathbf{i}}} } = {{\mathbf{FIM}}\left( {{\varvec{\upchi}},\left\{ {{\varvec{\uptheta}}_{\theta } , {\varvec{\uptheta}}_{{\eta {\text{i}}}} } \right\}} \right), } \\ { {\mathbf{Prior}} = {\varvec{\Omega}}^{ - 1} } \\ \end{array}$$

with *n* being the number of individual parameter sets sampled.

### Inclusion of IOV in the FIM_MAP_

#### MAP_occ_

The IOV was added to the individual FIM as an occasion deviation sampled per individual occasion from the prior IOV distribution **Π**. The prior IOV covariance matrix **Π** was utilized as occasion prior (Eq. 10).

10 $$\begin{array}{*{20}l} {{\mathbf{FIM}}_{{{\text{i}},{\text{MAP}}_{\text{occ}} }} = {{\mathbf{FIM}} }\left( {{\varvec{\upchi}}, \left\{ {{\varvec{\uptheta}}_{\theta } , {\varvec{\uptheta}}_{{\eta {\text{i}}}} , {\varvec{\uptheta}}_{\kappa 1,i} , {\varvec{\uptheta}}_{\kappa 2,i} , \ldots {\varvec{\uptheta}}_{\kappa m,i} } \right\}} \right)} \\ { {\mathbf{Prior}}_{{{\text{MAP}}_{\text{occ}} }} = \text{diag}\left( {{\varvec{\Omega}}^{ - 1} , {\varvec{\Pi}}_{1}^{ - 1} ,{\varvec{\Pi}}_{2}^{ - 1} , \ldots {\varvec{\Pi}}_{\text{m}}^{ - 1} } \right)} \\ \end{array}$$

where ${\varvec{\uptheta}}_{{\kappa {\text{ji}}}}$ is the vector of occasion deviations for the jth occasion of the ith individual, *m* is the number of occasions and **Π**_j_ == **Π**.

The inclusion of the occasion deviations in the MAP_occ_ approach is analogous to how the individual deviations are handled in the FIM_MAP_ (Eq. 8).

#### POP_occ_

The IOV was included in the individual FIM as an occasion variance term:

11 $$\begin{array}{*{20}l} {{\mathbf{FIM}}_{{{\text{i}},{\text{POP}}_{\text{occ}} }} = \mathbf{FIM}\left( {{\varvec{\upchi}}, \left\{ {{\varvec{\uptheta}}_{\theta } , {\varvec{\uptheta}}_{{\eta {\text{i}}}} } \right\}, {\varvec{\Pi}}} \right)} \\ {{\mathbf{Prior}}_{{{\text{POP}}_{\text{occ}} }} = \text{diag}\left( {{\varvec{\Omega}}^{ - 1} , \varvec{0_{v,v}} } \right)} \\ \end{array}$$

where **0**_**v,v**_ is the *v*-by-*v* zero matrix acting as prior for **П**, and *v* is the number of occasion effects in the prior population model. Using the first order (FO) approximation proposed by Retout and Mentré [17] the occasion variance contribution to the individual FIM can then be written [18]:

12 $$\begin{array}{*{20}c} {\mathop \sum \limits_{j = 1}^{m} \left( {\frac{{\partial {\text{f}}_{i} \left( . \right)|_{{\varvec{\kappa}_{x,i} }= 0} }}{{\partial\varvec{\kappa}_{i,j} }} \times {\varvec{\Pi}} \times \left( {\frac{{\partial {\text{f}}_{i} \left( . \right)|_{{\varvec{\kappa}_{x,i} }= 0} }}{{\partial\varvec{\kappa}_{i,j} }}} \right)^{'} } \right) } \\ {\text{where:}} \\ {{\text{f}}_{i} \left( . \right) = f\left( {{\varvec{\upchi}}_{\text{i}} , {\text{g}}\left( {{\varvec{\uptheta}}, {\varvec{\uptheta}}_{{\eta {\text{i}}}} , {\varvec{\upkappa}}_{{1,{\text{i}}}} , {\varvec{\upkappa}}_{{2,{\text{i}}}} \ldots ,{\varvec{\upkappa}}_{{{\text{m}},{\text{i }}}} } \right)} \right)} \\ \end{array}$$

As reference the designs were optimized without inclusion of IOV (using the FIM_MAP_ as is), either ignoring the known IOV (case *Omit*) or using a prior IIV distribution inflated with IOV (case *Inflate*). The latter mimic the model result from a study design neglecting the possibility to separate IIV and IOV.

### Design optimization

The models were implemented in the ODs software PopED version 2.13 [18] written in MATLAB (MATLAB v.7.12.0.635) using the FO approximation of the FIM. The prior FIM functionality was utilized to supply the prior IIV covariance matrix **Ω**. PopED supports IOV as a population random effect making the implementation of method POP_occ_ straightforward. The individual deviations vector ${\varvec{\uptheta}}_{{\eta {\text{i}}}}$ for the FIM calculation was drawn from **Ω** and reused for each model and optimization iteration to decrease the Monte Carlo error. The occasion parameter vectors for the MAP_occ_ method ${\varvec{\uptheta}}_{\kappa j,i}$ were sampled in the same manner. The expectation of the logarithm of the determinant of the interesting part of the FIM over the prior was employed as the criterion to be maximized (Ds optimality [7]):

13 $${\text{OFV}} = {\text{E}}_{{{\mathbf{Prior}}}} \left[ {\log \left( {\frac{{\left| {{\mathbf{FIM}}_{\text{i}} + {\mathbf{Prior}}} \right|}}{{\left| {{\mathbf{FIM}}_{{{\mathbf{i,uninteresting}} }} + {\mathbf{Prior}}} \right|}}} \right)} \right]$$

where **FIM**_**i,uninteresting**_ is the FIM for uninteresting parameters (defined below). For the MAP_occ_ criterion the occasion deviation parameters ${\varvec{\uptheta}}_{\kappa j,i}$ were taken as uninteresting while the POP_occ_ method was computed with the occasion variance parameters **Π** fixed. In all cases (Omit, Inflate, MAP_occ_, POP_occ_) the residual error variances, **Σ**, and the population parameters, ${\varvec{\uptheta}}_{\theta }$, were fixed.

The sampling schedules for the two test models were optimized using the PopED Random Search, Stochastic Gradient and Line search methods as described by Nyberg et al. [18]. Schedules of 3 or 6 sampling times over 36 h (three occasions) were investigated for the colistin PK model. The number of samples was selected as the smallest number needed to identify the η deviations in the absence of IOV and adding one extra sample per investigated occasion (3 and 3 + 3). Sampling was prohibited during and up to 15 min post infusion by setting the information to zero for samples placed in these intervals. Both CMS and colistin concentrations were assumed to be analysed at each time point. For the 1-COMP model a sampling schedule of 5 samples was optimized over 24 h, with no restriction in time. In order to investigate sampling clustering behaviour the number of samples were set to exceed the number needed to identify the η deviations in the absence of IOV.

### Standard error prediction

The predicted individual standard error (iSE) of the individual parameters (${\varvec{\uptheta}}_{{\eta {\text{i}}}}$) were computed as the square root of the diagonal of the inverse individual FIM plus the prior_:_

14 $${\varvec{iSE}}_{{{\mathbf{pred}}}} = \sqrt {{\text{diag}}\left( {{\mathbf{FIM}}_{\text{i}} + {\mathbf{Prior}}} \right)^{ - 1} }$$

where ${\varvec{iSE}}_{{{\mathbf{pred}}}}$ is the 1-by-*u* vector of predicted mean standard deviations of the *u* model η_EBE_s of individual i.

### Shrinkage prediction

Combes et al. [15] calculated the expected shrinkage (SH) in the η_EBE_s from the FIM_MAP_ and its prior according to:

15 $${\varvec{SH}}_{{{\mathbf{pred}}, {\mathbf{VAR}}}} = {\text{diag}}\left( { {\mathbf{FIM}}_{\text{MAP}}^{ - 1} \times {\mathbf{Prior}} } \right)$$

where *SH*_**pred,VAR**_ is the 1-by-*u* vector of predicted shrinkages of the *u* model η_EBE_s on a variance scale.

Equation 15 quantifies the information gain of the FIM_MAP_ compared to its prior; if no information is gained the FIM_MAP_ will be equal to its prior and the SH will be 1, conversely if much information is gained the FIM_MAP_ is large compared to its prior and the SH is low. The accuracy of the prediction is however dependent on how well the FIM_MAP_ reflect the individual information loss in the η_EBE_. The FIM_MAP_ may be substituted for the expected covariance matrix, i.e. the expectation of the inverse of the individual FIM plus its prior, which directly account for the individual contribution to the total SH as the inverse of the FIM is performed prior to summation. The predicted SH is then written:

16 $${\varvec{SH}}_{{{\mathbf{pred}}, {\mathbf{VAR}}}} = {\text{diag}}\left( {\left( {\frac{1}{\text{n}}\mathop \sum \limits_{{{\text{i}} = 1}}^{\text{n}} \left( {{\mathbf{FIM}}_{\text{i}} + {\mathbf{Prior}} } \right)^{ - 1} } \right) \times {\mathbf{Prior}} } \right)$$

The expression in Eq. 16 is corresponding to the expected shrinkage over the n sampled individual parameter sets. Commonly in pharmacometrics (e.g. [2]) shrinkage is presented on the standard deviation scale, Eq. 16 then becomes:

17 $${\varvec{SH}}_{{{\mathbf{pred}}}} = 1 - \sqrt { {\text{diag}}\left( {{\mathbf{I}} - \left( {\frac{1}{\text{n}}\mathop \sum \limits_{{{\text{i}} = 1}}^{\text{n}} \left( {{\mathbf{FIM}}_{\text{i}} + {\mathbf{Prior}} } \right)^{ - 1} } \right) \times {\mathbf{Prior}} } \right)}$$

where *SH*_**pred**_ is the vector of predicted shrinkages of the *u* model η_EBE_s on a standard deviation scale.

### Computation time

The computation time for each method was assessed as the mean estimation time of 100 individual FIM calculations on an Intel i7 2.7 GHz machine running MATLAB v.7.12.0.635 on Windows 7.

### Design evaluation

The performance of the designs was assessed via 10,000 Monte Carlo simulation–MAP estimation procedures of the designs using the full population model implemented in NONMEM 7.3 (Colistin PK: ADVAN5, FOCEI, 1-Compartment IV-bolus: ADVAN1, FOCEI). The simulation/estimation was carried out using the SSE functionality in PsN v.4.0.1 [19] with η_EBE_s estimated by setting the MAXEVAL = 0 and MCETA = 1000 option in NONMEM. The individual SEs (${\varvec{iSE}}_{ \exp }$) of the EBEs were obtained as the square root of the individual predicted η_EBE_ variance from the NONMEM 7.phi file, see Kang et al. [20].

The coefficient of determination (R^2^) between the simulated η and the MAP estimated η_EBE_ was used as a measure of re-estimation precision for the population and obtained as:

18 $$R_{r }^{2} = 1 - \frac{{\mathop \sum \nolimits_{i = 1}^{n} \left( {\eta_{{EBE_{i,r} }} - \eta_{i,r} } \right)^{2} }}{{\mathop \sum \nolimits_{i = 1}^{n} \left( {\mathop {\bar{\eta}_{r}} - \eta_{i,r} } \right)^{2} }}$$

where *R*_*r*_^2^ is the R^2^ of the rth *η*_*EBE*_, *η*_i,r_ is the rth simulated individual deviation for individual *i*, $\mathop {\bar{\eta }_{r}}$ is the mean of the rth simulated individual deviation (in the ideal case equal to 0), $\eta_{EBE_{i,r}}$ is the corresponding EBE, and *n* is the number of simulated individuals (here 10,000). Note that negative R^2^ values are possible if the variance of the difference between the simulated and the estimated deviations is larger than the variance of the simulated values, i.e. the precision of the re-estimation is worse than what would be achieved if each value was set to the mean.

The η-shrinkage was calculated as:

19 $$SH_{r} = 1 - \frac{{SD\left( {\eta_{{EBE_{r} }} } \right)}}{{\omega_{r} }}$$

where *SH*_r_ is the shrinkage of the rth *η*_*EBE*_ on standard deviation scale and *ω*_r_ is the prior standard deviation.

## Results

### Designs

**Fig. 1:**
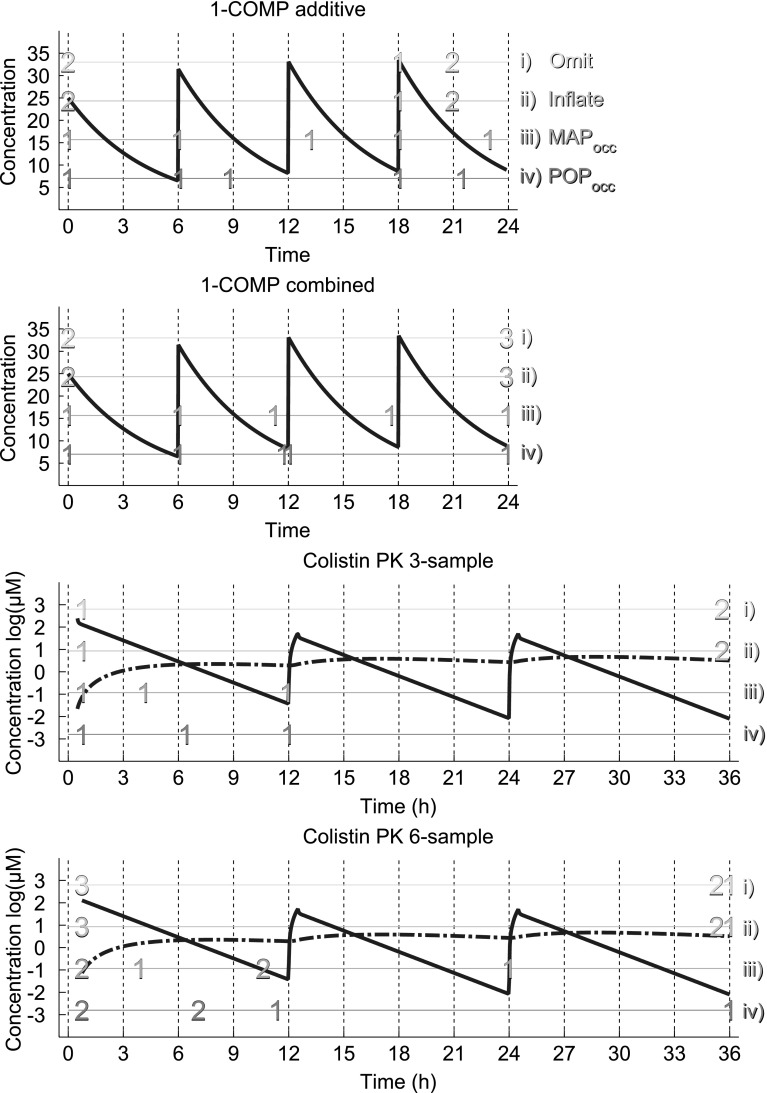
PK profiles of models 1-COMP (*top*) and Colistin PK (*middle and bottom*, Colistin *solid line*, CMS *dashed line*) and sampling schedules for the Omit, Inflate, MAP_occ_, and POP_occ_ (from *top* to *bottom* in each panel) with the number of samples per time point indicated on *horizontal lines*. For all models samples are assumed to be taken post-dose in the respective occasion

The two cases ignoring the IOV in the optimization (Omit and Inflate) resulted in identical 5-sample designs for the 1-COMP additive error model (Fig. 1, top panel) placing duplicate samples at Cmax in the first occasion (first dosing interval) and three samples in the last occasion, a single sample at Cmax and a duplicate sample in the middle. The MAP_occ_ method allocated single samples across all occasions (two samples in the last occasion) while POP_occ_ sampled the first, third and last occasion. When the proportional residual error was added the Omit and Inflate cases again resulted in identical designs with duplicate sampling of the Cmax of the first occasion and triplicate sampling of the Cmin of the last occasion. MAP_occ_ and POP_occ_ placed samples early and/or late in each occasion (Fig. 1, 2nd panel).

For the Colistin PK model the Omit and Inflate methods also resulted in identical designs placing a single sample at the first available time of the first occasion and duplicate samples at the end of the last occasion for the three sample design (Fig. 1, 3rd panel). For the six-sample design triplicate samples were included in the first occasion plus three clustered samples at the end of the last occasion (Fig. 1, bottom panel). In contrast the MAP_occ_ and POP_occ_ methods placed single samples at the start, middle and end of the first occasion for the three sample design. For the six sample design the MAP_occ_ method added duplicate samples at the start and end of the first occasion and a single sample at the end of the second occasion while the POP_occ_ added duplicate samples to the start and middle of the first occasion and a single sample at the end of the last occasion.

The general layout of the designs produced by each method was robust to the random seed used to initiate the optimization. E.g. for the 3 sample Colistin PK design an alternate deviation vector shifted the sampling times by ~1 % of the dosing interval.

### Re-estimation

As the Omit and Inflate cases provided identical designs the re-estimation performance will only be presented for the Omit design.

**Fig. 2:**
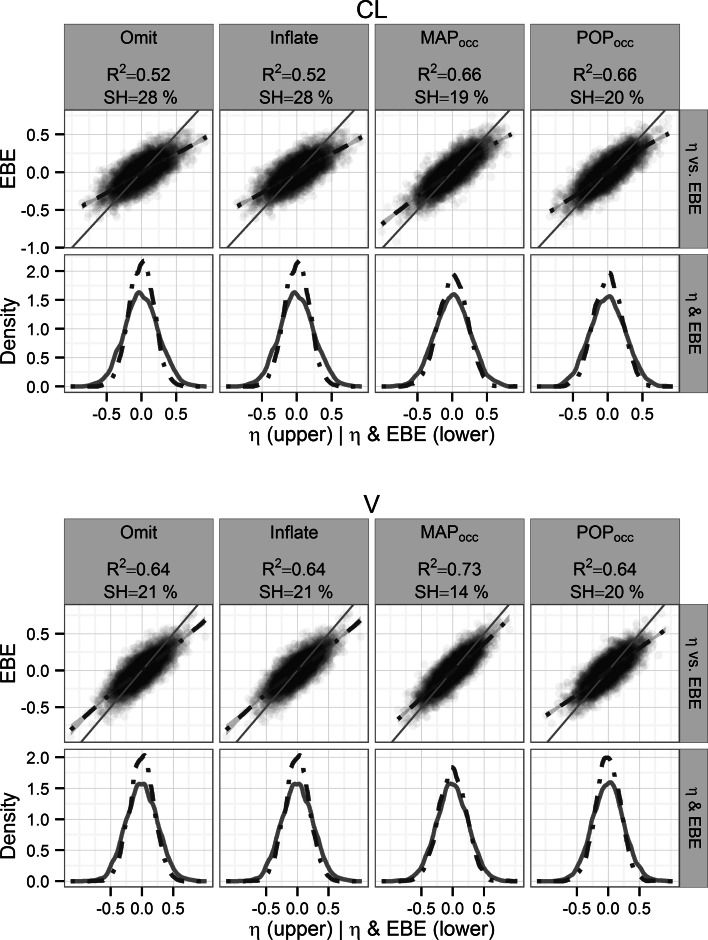
Re-estimation performance per design for the 1-COMP model with additive error. η_CL_ (*top panel*) and η_V_ (*bottom panel*), the coefficient of determination with respect to the simulated η (R^2^) and the η-shrinkage (SH %) of the re-estimation are given. *Upper row* Simulated η versus corresponding η_EBE_ with the line of identity (*solid*) and a smooth of the intercepts (*dashed*). *Lower row* Distribution of simulated η (*solid line*) and corresponding η_EBE_ (*dashed line*)

**Fig. 3:**
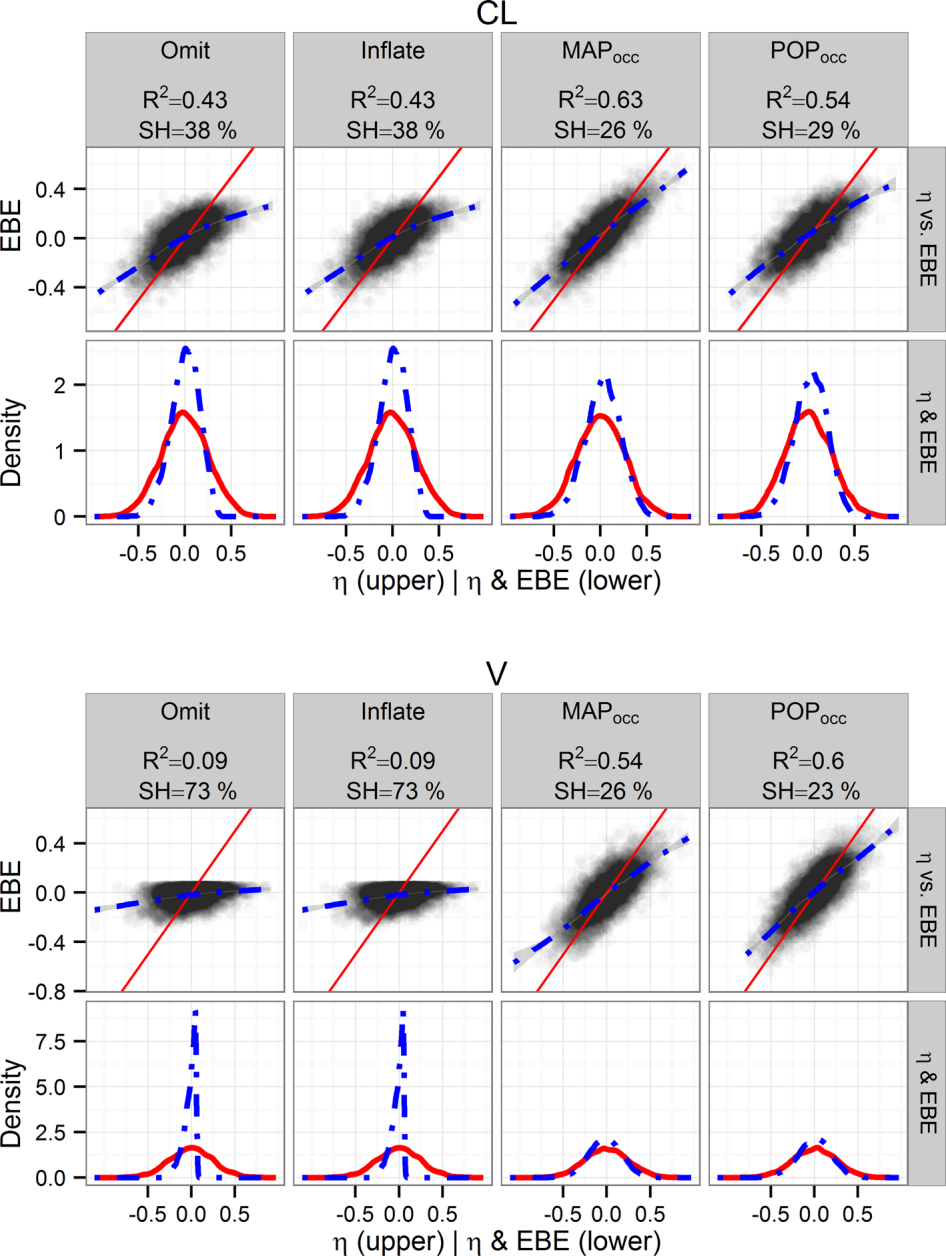
Re-estimation performance per design for the 1-COMP model with combined error. η_CL_ (*top panel*) and η_V_ (*bottom panel*), the coefficient of determination with respect to the simulated η (R^2^) and the η-shrinkage (SH %) of the re-estimation are given. *Upper row* Simulated η versus corresponding η_EBE_ with the line of identity (*solid*) and a smooth of the intercepts (*dashed*). *Lower row* Distribution of simulated η (*solid line*) and corresponding η_EBE_ (*dashed line*)

For the 1-COMP additive residual error model the baseline Omit design performed the worst for both parameters (η_CL_: R^2^ = 0.52 and SH = 28 %, η_V_: R^2^ = 0.64 and SH = 21 %) and MAP_occ_ performed the best (η_CL_: R^2^ = 0.66 and SH = 19 %, η_V_: R^2^ = 0.73 and SH = 14 %) (Fig. 2). POP_occ_ had intermediate performance (η_CL_: R^2^ = 0.66 and SH = 20 %, η_V_: R^2^ = 0.64 and SH = 20 %). Similar results were found for the combined residual error model where the Omit design also was the worst (η_CL_: R^2^ = 0.43 and SH = 38 %, η_V_: R^2^ = 0.09 and SH = 73 %) while MAP_occ_ (η_CL_: R^2^ = 0.63 and SH = 26 %, η_V_: R^2^ = 0.54 and SH = 26 %) and POP_occ_ (η_CL_: R^2^ = 0.59 and SH = 24 %, η_V_: R^2^ = 0.60 and SH = 23 %) had the best performance for η_CL_ and η_V_ respectively (Fig. 3).

**Fig. 4:**
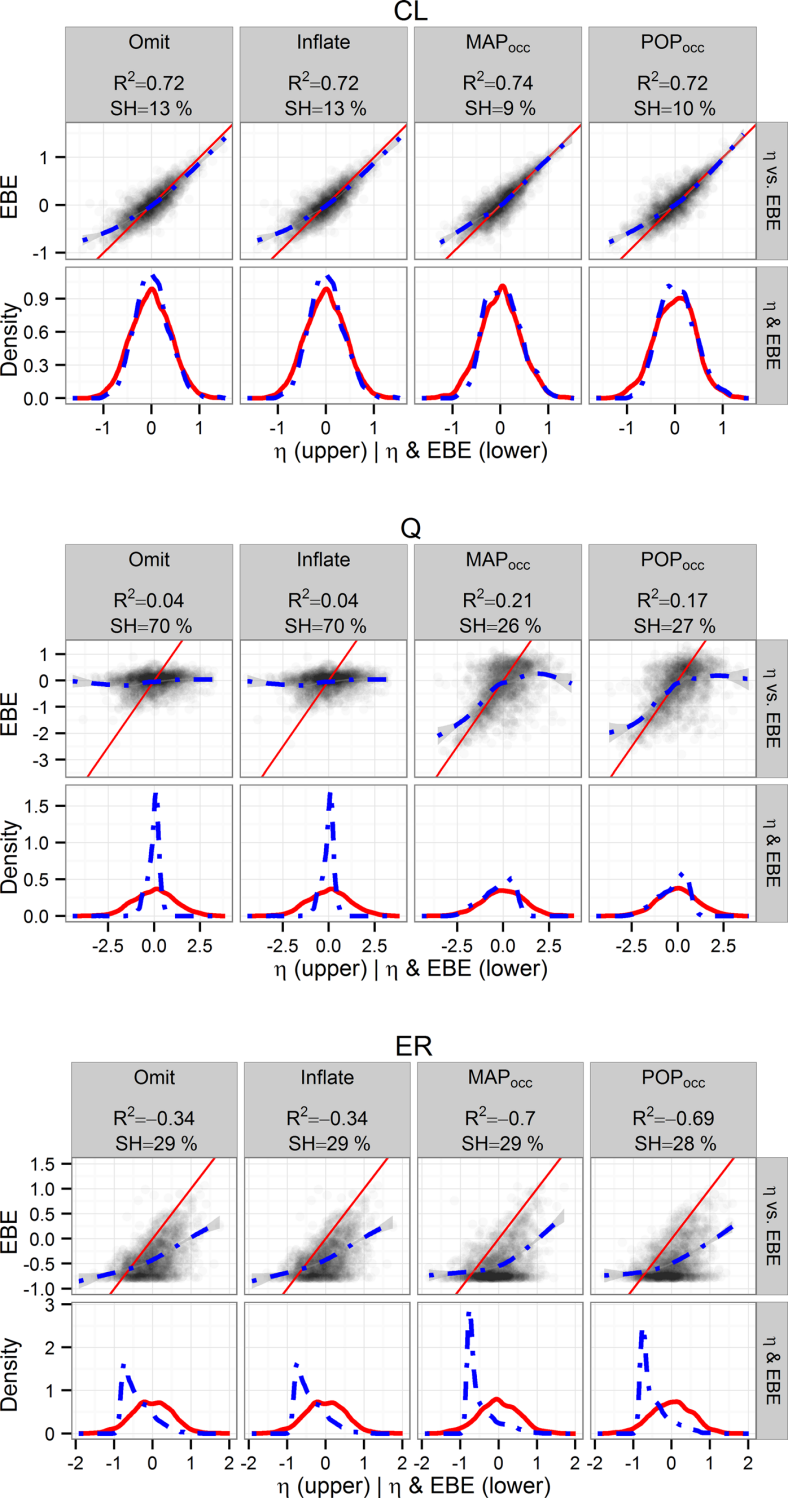
Re-estimation performance per design for the Colistin PK 3-sample model η_CL_ (*top panel*), η_Q_ (*middle panel*) and η_ER_ (*bottom panel*), the coefficient of determination with respect to the simulated η (R^2^) and the η-shrinkage (SH %) of the re-estimation are given. *Upper row* Simulated η versus corresponding η_EBE_ with the line of identity (*solid*) and a smooth of the intercepts (*dashed*). *Lower row* Distribution of simulated η (*solid line*) and corresponding η_EBE_ (*dashed line*)

**Fig. 5:**
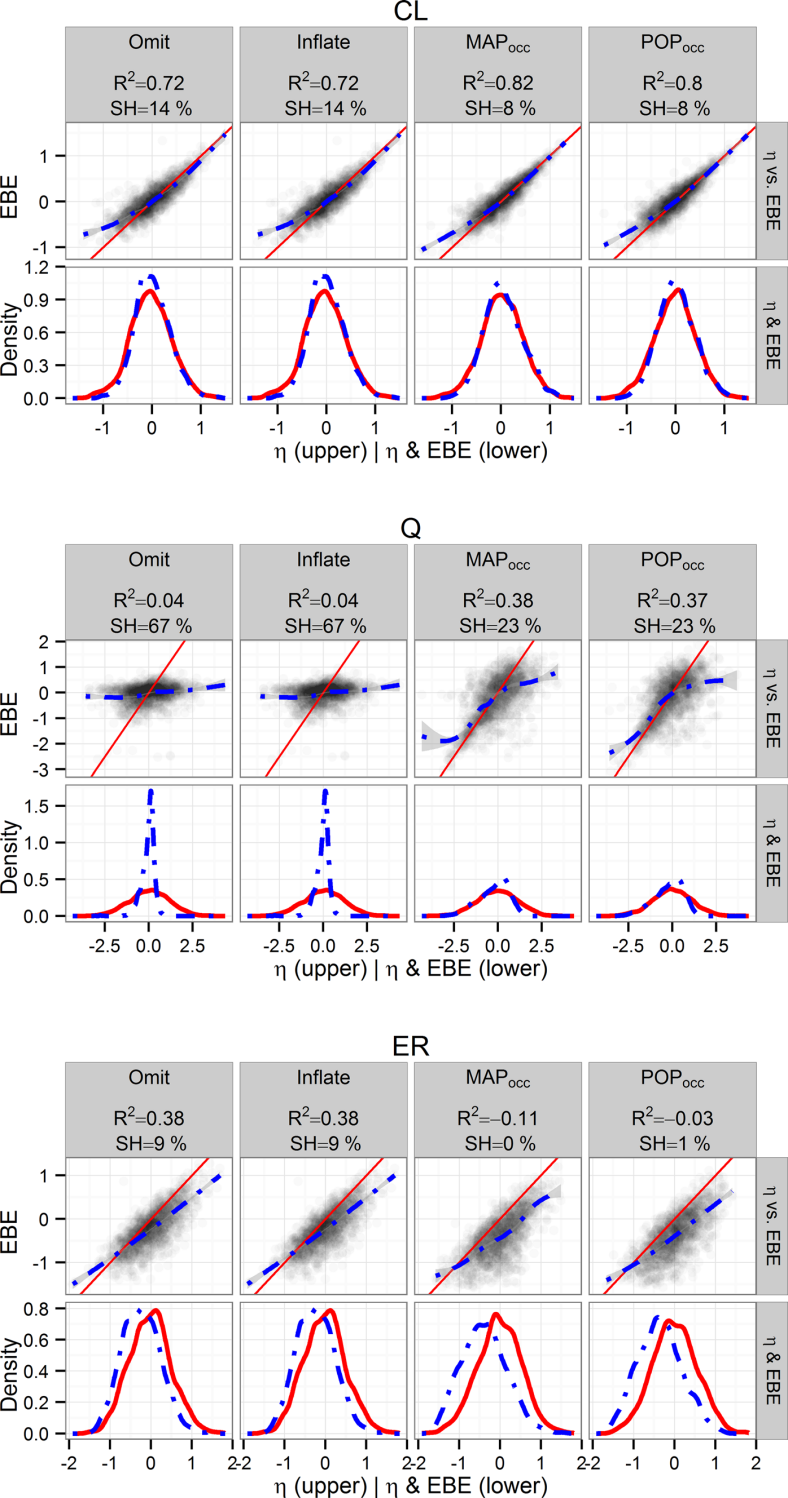
Re-estimation performance per design for the Colistin PK 6-sample model η_CL_ (*top panel*), η_Q_ (*middle panel*) and η_ER_ (*bottom panel*), the coefficient of determination with respect to the simulated η (R^2^) and the η-shrinkage (SH %) of the re-estimation are given. *Upper row* Simulated η versus corresponding η_EBE_ with the line of identity (*solid*) and a smooth of the intercepts (*dashed*). *Lower row* Distribution of simulated η (*solid line*) and corresponding η_EBE_ (*dashed line*)

For the Colistin PK model 3 sample designs the η_CL_ was re-estimated with similar accuracy and precision (R^2^ ≥ 0.72) for all methods (Fig. 4, upper panel), while the SH was lower for MAP_occ_ and POP_occ_ methods (SH ≤ 10 %) compared to Omit (SH = 13 %). MAP_occ_ achieved the highest precision (R^2^ = 0.74) and the lowest SH (10 %). As the number of available samples was increased to six the precision was unchanged for Omit while the SH remained similar (R^2^ = 0.72, SH = 14 %) (Fig. 5, upper panel). In contrast, for the MAP_occ_ and POP_occ_ methods the precision increased and SH decreased when the number of samples was doubled (R^2^ ≥ 0.82, SH ≤ 8 %). POP_occ_ performed the best (R^2^ = 0.8, SH = 8 %).

The η_Q_ parameter was less well re-estimated (Figs. 4, 5 middle panels) compared to the η_Cl_ parameter and the difference between the re-estimation performance of Omit (3 samples: R^2^ = 0.04, SH = 70 %, 6 samples: R^2^ = 0.04, SH = 67 %) and the MAP_occ_ and POP_occ_ methods (3 samples: R^2^ ≥ 0.17, SH ≤ 27 %, 6 samples: R^2^ ≥ 0.37, SH ≤ 23 %) was higher. Overall, the MAP_occ_ designs had the best performance (3 samples: R^2^ = 0.21, SH = 26 %, 6 samples: R^2^ = 0.38, SH = 23 %).

None of the 3-sample designs for the Colistin PK model were able to re-estimate the η_ER_ parameter with R^2^ values above 0, and although SH was not high (<29 % for all designs) the distributions were highly skewed (Fig. 4, bottom panel). When the number of available samples was increased to 6 the Omit design performed best in terms of R^2^ (0.38) whereas MAP_occ_ was best in terms of SH (0 %), however all designs provided very low SH (<9 %) (Fig. 5, bottom panel).

### Correspondence between PopED and NONMEM iSE

**Fig. 6:**
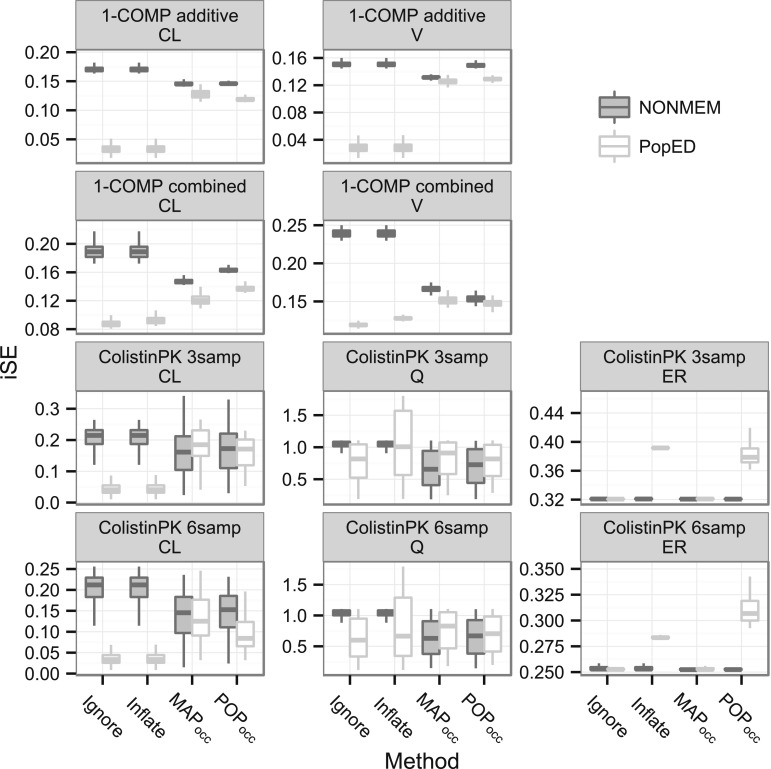
Boxplots (1st, 2nd, 3rd quartile + whiskers) of the distribution of individual SE (iSE) per parameter and design from PopED (SE of *θ*_*ik*_) and NONMEM (SE of *η*_*EBE*_). The *upper* whisker extend to the highest value that is within 1.5 * IQR of the 3rd quartile, where IQR is the inter quartile range. The *lower* whisker extends to the lowest value within 1.5 * IQR of the 1st quartile

The correspondence between the individual SE (iSE) for the η-parameters as given by the PopED prediction and NONMEM evaluation are illustrated in Fig. 6. The agreement between the iSE was evaluated both in terms of size as given by the median, and in terms of spread in the population as given by the inter quartile range (IQR).

For the 1-COMP model the correspondence of the median iSE was poor for the Omit and Inflate scenarios and best for the MAP_occ_ method while the POP_occ_ method had slightly larger differences in the median iSE. The IQR was small for all designs with a slight tendency of overprediction observed for Inflate, Omit, and MAP_occ_ for both the η_CL_ and η_V_.

For the Colistin PK 3 and 6 sample designs, the Omit and Inflate scenarios underpredicted both the iSE median and IQR for the η_CL_ and η_Q_ parameters. For η_ER_ Omit accurately predicted the observed median and the (very small) IQR while Inflate overpredicted the median but predicted the IQR well. MAP_occ_ predicted the median and IQR of the iSE well for all parameters. For the 3 sample designs POP_occ_ accurately predicted the median and IQR of η_CL_ and η_Q_ while for the 6 sample design they accurately predicted the η_Q_ iSE and IQR. For both the 3 and 6 sample designs POP_occ_ overpredicted the η_ERE_ iSE median and IQR.

### Correspondence between predicted and observed shrinkage

**Fig. 7:**
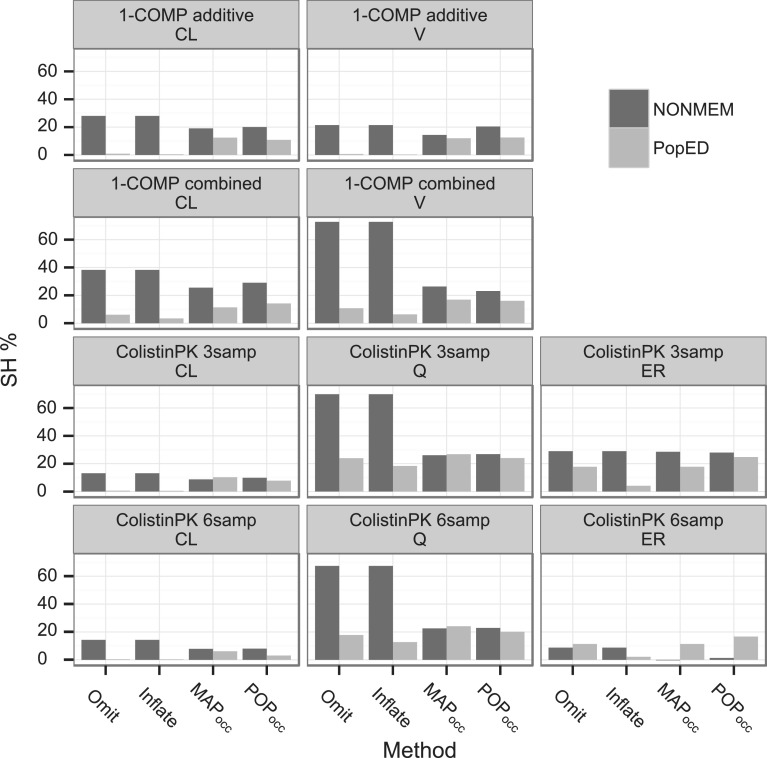
The predicted SH (PopED) per parameter and design (using the respective methods) compared with the re-estimated SH (NONMEM) (using the full model)

The cases Omit and Inflate strongly underpredicted the observed SH for all models and parameters except for the Colistin PK η_ER_ for which Omit moderately underpredicted for the 3 sample design and overpredicted for the 6 sample design (Fig. 7). For the 1-COMP model the MAP_occ_ and POP_occ_ methods tended to moderately underpredict the SH for both parameters. For the Colistin PK model 3 sample design MAP_occ_ accurately predicted the SH in the η_Cl_ and η_Q_ distributions and slightly underpredicted the η_ER_ SH, while POP_occ_ accurately predicted the SH of all three parameters. As the information increased with the addition of three samples in the 6 sample design the MAP_occ_ and POP_occ_ methods underpredicted the η_CL_ SH (MAP_occ_ performing best, POP_occ_ slightly worse), accurately predicted the η_Q_ SH and underpredicted the η_ER_ SH.

### Runtimes

**Fig. 8:**
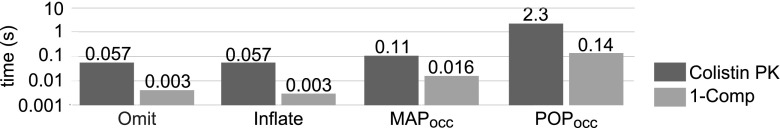
Runtime on a 2.7 GHz intel i7 machine for one FIM calculation for the two models respectively

Relative to the base FIM_map_ runtime (Omit and Inflate) the runtime for one PopED FIM calculation for methods MAP_occ_ and POP_occ_ of the 3-sample colistin PK design were 1.8 and 38 times longer, respectively, and for the 1-COMP additive residual error model 4.0 and 46 times longer, respectively (Fig. 8).

## Discussion

The ability to optimize for individual parameter precision in the presence of IOV could be important to improve the ability to design studies reliant on precise individual parameter estimates, e.g. feedback dose individualization. However also studies with the aim of describing population characteristics could be facilitated as many covariate model building techniques utilize EBEs [3] and are thus inherently reliant on precise individual parameter estimates [21]. The level of IOV limits the applicability for feedback dose individualization [22] and is therefore important to consider, but will also affect the precision with which individual deviations may be estimated. OD with the aim of providing precise individual parameter estimates for models including IOV has been investigated previously by e.g. Nguyen et al. [23] where a standard population D-optimality method was used. However this is to the authors’ knowledge the first effort to include handling of IOV in individual OD using MAP based FIM.

The two cases of ignoring IOV in OD for individual parameters resulted in identical designs. In contrast, including the IOV as a fixed effect per occasion (MAP_occ_) or as an occasion random effect (POP_occ_) markedly shifted the design. The result that Inflate did not shift the design relative to Omit may be anticipated as it does not convey any information penalty for designs that cannot discriminate between individual and occasion deviations. In contrast, MAP_occ_ and POP_occ_ both treat the occasion deviations as modeled variables. Hence lack of information to discriminate between the individual and occasion deviations directly impacts the expected parameter precision.

For the 1-COMP model with an additive residual error this resulted in designs with a wide spread of samples over the available occasions for methods MAP_occ_ and POP_occ_, maximizing the ability to discriminate between individual and occasion deviations. In contrast Omit produced clustered samples at the first and last occasion. When the proportional residual error was introduced the emphasis was shifted for all methods from sampling at high to low concentrations in an effort to minimize the noise. The general sampling strategy was however similar to the additive residual error case with Omit concentrating samples to two time points while MAP_occ_ and POP_occ_ spread single samples across all available occasions. When the impact of these two strategies was evaluated it was apparent that the MAP_occ_ and POP_occ_ designs provided higher accuracy and less shrinkage and bias in the η_EBE_ estimates compared with Omit, regardless of the residual error model. In general the performance of MAP_occ_ was slightly superior to POP_occ_.

For the Colistin PK model the POP_occ_ and MAP_occ_ 3-sample designs concentrated all samples to the first occasion while Omit also in this case sampled the first and last occasion. The concentration of samples to one occasion for MAP_occ_ and POP_occ_ is contrary to the 1-COMP design (irrespectively of the residual error model) and the expectation that maximizing the number of sampled occasions would maximize the possibility of separating occasion and individual deviations. The reason is the structure of the random effects in the Colistin PK model as the occasion and individual deviations are linearly independent with respect to the model response. This make it possible to separate the occasion and individual deviations on any one occasion (Eqs. 2, 3, 4). It would thus be of advantage to sample few and early occasions, as samples taken at late occasions would be influenced by deviations from early occasions carried forward in the PK profile. Additionally for colistin there is a larger range of concentrations in the first occasion providing information on the volume of distribution. Three samples was sufficient to saturate the first occasion for both MAP_occ_ and POP_occ_ and the additional three samples of the 6-sample design resulted in the addition of one new sampling point at a later occasion as well as duplicated samples. In contrast, for the Omit design the added samples simply resulted in additional clustered samples as the support points for the model without IOV were already occupied.

For the Colistin PK model the re-estimation performance of the MAP_occ_ and POP_occ_ designs was superior to the Omit design except for the η_ER_ parameter (Figs. 4, 5) for which the MAP_occ_ and POP_occ_ designs resulted in biased η_EBE_ distributions. The systematic negative bias of the re-estimated η_ER_ may be due to an inability to sufficiently separate the residual error and the occasion deviations leading to an underestimation of the residual error variance. In contrast the multiplicity of the sampling points in the Omit design allowed more precise residual error characterization and hence a better ability to determine the η_ER_.

The effect of sampling in a limited number of occasions for the two models was investigated by placing rich sampling (>1 sample/h) in either the first occasion or in all available occasions for the Colistin PK model and in either the first, first and second, or all occasions for the 1-COMP model. These designs were evaluated by simulation and MAP reestimation in NONMEM (results not shown). For the Colistin PK model rich sampling in one occasion was sufficient to estimate all η_EBE_s with adequate precision and shrinkage (R^2^ ≥ 0.77, SH ≤ 15 %) which was moderately improved by rich sampling in all three occasions (R^2^ ≥ 0.88, SH ≤ 11 %). As reflected in the 3 and 6 sample designs for methods POP_occ_ and MAP_occ_, this result confirms that due to linear independence of the random effect parameters most information is available in the first occasion for the Colistin PK model but that additional information may be gained by adding samples in later occasions. For the 1-COMP model there was a clear gain in precision and decrease in shrinkage as the number of sampled occasions was increased from the first (R^2^ ≥ 0.48, SH ≤ 31 %), to the first and second (R^2^ ≥ 0.63, SH ≤ 21 %), and finally to all four occasions (R^2^ ≥ 0.74, SH ≤ 14 %). Again this result confirms the sampling strategy of methods POP_occ_ and MAP_occ_. In addition, for a model where IIV and IOV variances are added to the same fixed effect parameter, the result illustrates that the EBEs will always be subject to shrinkage when a finite number occasions are sampled. The iSE prediction was generally good both in terms of size and spread for the MAP_occ_ and POP_occ_ methods, albeit with a tendency of negative bias (Fig. 6). In contrast methods Omit and Inflate behaved poorly, however only Omit and MAP_occ_ accurately predicted the Colistin PK η_ER_ iSE. A reason for the negative bias of the predicted iSE may be that the prediction is based on the symmetrical and centered **Ω** distribution while the re-estimated iSE is based on the actual ηEBE from NONMEM. In an effort to increase the quality of the NONMEM η_EBE_ the MCETA option available in version 7.3 was used by which additional initial estimates for the EBEs are tested.

The SH prediction by the MAP_occ_ and POP_occ_ methods for the 1-COMP model was in the range of accuracy demonstrated by Combes et al. [15] and excellent for Colistin PK 3 sample design where the MAP_occ_ method predicted the η_ER_ SH with high accuracy. For the 6 sample design the predictions deteriorated, possibly due to a larger discrepancy between the observed and predicted η-variances. The Omit and Inflate methods failed to predict the SH for all models and parameters except the Colistin PK η_ER_, the same trend may be noted for the iSE. The finding that the SH prediction was worse for the simpler 1-COMP model may be due to that the individual and occasion deviations in this model are added to the same parameter, making the separation of these variances harder.

As expected, ignoring the IOV was fastest in terms of computational effort followed by MAP_occ_ by an increase roughly proportional to the number of added parameters. The POP_occ_ method was associated with a pronounced increase in computational effort due to the need to linearize around the occasion random effects in addition to the residual error [18]. The differences in computational effort are not expected to be sensitive to the structure of the IIV variances or the residual error model since these are the same for the different methods. However the number of occasions may potentially shift the computational effort of POP_occ_ and MAP_occ_ as the latter needs one new set of parameters added per occasion whereas POP_occ_ instead has to linearize over the additional occasions.

The clustering of samples (as observed for several of the designs presented in this work) is a common behavior in design optimization when the design is saturated, i.e. that the support points needed to identify the model parameters are populated, and any additional samples will be focused on improving the signal-to-noise of the measurement [24]. The gain in parameter precision is however dependent on the assumption that the errors of duplicate samples taken at the same time are uncorrelated, an assumption that is unlikely to hold for real data. The clustering behavior can be avoided by acknowledging the inter dependence of samples in the model building and design optimization [24], or by empirically spreading the sampling clusters.

In this work it was only considered to add the occasion variances as fixed parameters to the POP_occ_ method as this reflect the same assumption as for the MAP_occ_ method; namely that the variances of the occasion deviations are known from the prior. The POP_occ_ method could be further expanded to consider the occasion random effect as unfixed (set as interesting or uninteresting) in the optimization. These alternative implementations were evaluated but differences were found to be small compared with the differences between the tested methods, both in the produced design and the predictability of the method (result not shown). Additionally only FO based FIMs were evaluated, linearization of the model around the conditional estimates of the occasion deviations (FOCE) may have improved the performance of the POP_occ_ method but would have severely increased the run times. While the influence of the balance between IIV, IOV, and residual error variances on the ability to precisely estimate η_EBE_s have not been investigated here it is likely that higher degrees of within subject variability (IOV and residual error) would limit the precision of which the individual deviation parameters may be estimated. However, in such a situation the η_EBE_s are expected to be of less value for feedback dose individualization or model diagnostics. We believe the results are generalizable in the sense that large IOV needs to be considered in the design of studies aiming to estimate η_EBE_s. The exact sampling patterns are however expected to be sensitive to differences in model structure and random effect levels.

## Conclusions

Two methods were formulated and applied to account for IOV in the optimization for maximum precision in individual parameters and evaluated against two scenarios of ignoring the IOV. Directly accounting for IOV resulted in designs markedly different from those suggested when ignoring the IOV, with large gains in the precision of the estimated individual deviation. In addition both methods (MAP_occ_ and POP_occ_) predicted the observed iSE and SH well. In contrast, ignoring IOV, either by omitting known IOV or by failing to separate IIV and IOV, led to overly optimistic shrinkage and precision predictions, and lower precision in the estimated individual parameters. While differences between MAP_occ_ and POP_occ_ were slight, both in the produced design and terms of predictability, MAP_occ_ was computationally much faster in the studied cases. MAP_occ_ is also attractive compared to POP_occ_ as the occasion deviations are handled analogously to the individual deviations in the FIM_MAP_. The POP_occ_ method is however easier to implement in PopED and could be advantageous if the number of occasions is large or if it is suitable to consider the IOV distribution of interest to estimate. Based on this work the authors would generally recommend the use of method MAP_occ_ in OD for individual parameter estimates in the presence of IOV.
